# Optimal crosstalk suppression in multicore fibers

**DOI:** 10.1038/s41598-019-51854-x

**Published:** 2019-10-31

**Authors:** B. Jaramillo Ávila, J. M. Torres, R. de J. León-Montiel, B. M. Rodríguez-Lara

**Affiliations:** 1 CONACYT-Instituto Nacional de Astrofísica, Óptica y Electrónica, Calle Luis Enrique Erro No. 1. Sta. Ma. Tonantzintla, Pue., C.P. 72840 Mexico; 2 Instituto de Física, Benémerita Universidad Autónoma de Puebla, Apdo. Postal J-48, Puebla, 72750 Mexico; 3 Instituto de Ciencias Nucleares, Universidad Nacional Autónoma de México, Apartado Postal 70-543, 04510 Cd. Mx., Mexico; 4 Tecnologico de Monterrey, Escuela de Ingeniería y Ciencias, Ave. Eugenio Garza Sada 2501, Monterrey, N.L. 64849 Mexico; 5 Instituto Nacional de Astrofísica, Óptica y Electrónica, Calle Luis Enrique Erro No. 1, Sta. Ma. Tonantzintla, Pue., CP 72840 Mexico

**Keywords:** Other photonics, Optics and photonics

## Abstract

We study propagation in a cyclic symmetric multicore fiber where the core radii randomly fluctuate along the propagation direction. We propose a hybrid analytic-numerical method to optimize the amplitude and frequency of the fluctuations that suppress power transfer between outer and inner cores. This framework allows us to analytically find noise amplitude parameters that optimally suppress crosstalk. Our predictions are confirmed by numerical experiments using finite difference beam propagation methods for realistic C-band fibers. The analytic part of our method is general, provides the optimum fluctuation amplitude independent of the array geometry, as long as normal modes can be calculated. It works for both correlated and uncorrelated fluctuations allowing its use for any given optical system described by coupled mode theory.

## Introduction

Multi-core fibers provide high-capacity optical transmission but dense packing induces crosstalk between cores affecting space division multiplexing^1–4^. Quasi-homogeneous structures induce crosstalk suppression via fluctuations in parameters and materials^5,6^. Transverse random fluctuations, constant along the propagation axis, produce transverse Anderson localization of light in otherwise periodic structures^7–10^. In contrast, introducing fluctuations that vary in the propagation direction produces faster than ballistic beam expansion, in a process similar to the so-called hyper-transport of light^11^ or environment-assisted quantum transport^12–17^.

We provide a theoretical description of experimental crosstalk suppression in a cyclic array of homogeneous cores with independent random fluctuations in core radii along the propagation direction. We focus on two experimental platforms, multicore fibers^5,6^ and laser inscribed waveguide arrays^16,18^. Random fluctuations are described by their statistical distribution, amplitude and frequency, that is, the strength and the number of variations per unit of length. Our aim is to optimize these parameters using hybrid analytic-numerical methods to gain insight of the underlying processes. In the following, we provide an analytic framework to find the fluctuation amplitude that produces optimal crosstalk suppression. Our approach is based on coupled mode theory and first-order perturbation theory. This allows us to find moderate-noise regions that produce optimal crosstalk suppression. There, increasing the noise amplitude does not increase crosstalk suppression. As an example, we use uniform random fluctuations which allow us to neglect all but the second moment of the random distribution. Then, we numerically calculate the optimal variation frequency for these fluctuation amplitudes. We compare our results with a numerical experiment using finite difference beam propagation methods with parameters from experimental devices in either multicore fibers or laser inscribed waveguide arrays.

## Results

### Model

**Figure 1:**
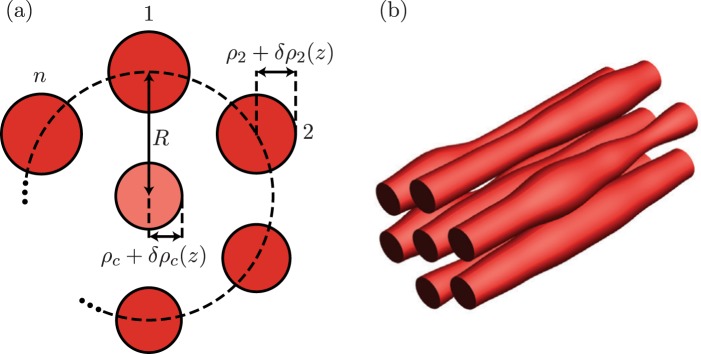
(**a**) Cyclic symmetric multi-core fiber cross-section and (**b**) sketch for independent random variations in core radii.

Our subject is a cyclic, multicore fiber composed by *n* + 1 single-mode cores whose radii randomly varies along the propagation direction, *r*_*j*_(*z*) = *ρ*_*j*_ + *δρ*_*j*_(*z*) with *j* = 1, …, *n*, *c*. All external cores have the same reference radii, *ρ*_1_ = … = *ρ*_*n*_; the central core may have a different one, *ρ*_*c*_, Fig. 1. These fluctuations are inevitable during fabrication. They are usually kept well below the 2% mark and they can be introduced in a controlled manner above this threshold^6^. Small, smooth, and well-behaved variations induce slight deformations on the localized LP_01_ field modes at each core, as well as negligible back-propagation and power loss, and we can use coupled mode theory,

1 $$-i\frac{{\rm{d}}}{{\rm{d}}z}\overrightarrow{ {\mathcal E} }(z)=M(z)\cdot \overrightarrow{ {\mathcal E} }(z),$$

to describe the propagation of just the complex mode amplitudes, $\overrightarrow{ {\mathcal E} }(z)={({ {\mathcal E} }_{1}(z),\ldots ,{ {\mathcal E} }_{n}(z),{ {\mathcal E} }_{c}(z))}^{T}$. The fluctuations induce variations in both the effective propagation and coupling constants, but the relative variation of the effective propagation constant is at least two orders of magnitude larger than that of the coupling constant. Thus, we neglect the effect of these variations on the coupling constants,

2 $$M(z)=(\begin{array}{cccccc}{b}_{1} & {g}_{1} & \cdots  & {g}_{2} & {g}_{1} & {g}_{c}\\ {g}_{1} & {b}_{2} & \ddots  & \cdots  & {g}_{2} & {g}_{c}\\ \vdots  & \ddots  & \ddots  & \ddots  & \ddots  & \vdots \\ {g}_{2} & \cdots  & {g}_{1} & {b}_{n-1} & {g}_{1} & {g}_{c}\\ {g}_{1} & {g}_{2} & \cdots  & {g}_{1} & {b}_{n} & {g}_{c}\\ {g}_{c} & {g}_{c} & \cdots  & {g}_{c} & {g}_{c} & {b}_{c}\end{array}),$$

with *b*_*j*_ ≡ *b*_*j*_(*z*) = *β*_*j*_ + *δβ*_*j*_(*z*), with *j* = 1, …, *n*, *c*. Here, all the external cores have the same reference propagation constant, *β*_1_ = … = *β*_*n*_, and the central core may have a different one, *β*_*c*_. The coupling constants are independent of the propagation direction, *z*. In the following, our numerics will refer to telecomm C-band, 1550 nm, with standard silica fibers, *n*_*c*_ = 1.4479 and *n*_*cl*_ = 1.4440. The reference radii and center-to-center separation are *r*_1,…,*n*,*c*_ = 4.5 *μ*m and *R* = 15 *μ*m, in that order. They yield approximated single-mode propagation constant *β* = 5.85975 × 10^6^ rad/m and intercore couplings *g* = 256.636 rad/m. This model also describes laser inscribed waveguide arrays where variations in the propagation constants are related to the radii and refractive index of individual waveguides, which can be controlled by the spot size and writing speed of the system. Reported controlled random radii variations are in the 5% range for multicore fibers^5,6^ and for femtosecond laser written photonic circuits the half-maximum reported variation in the refraction index is 3 × 10^−4^ ^18^. It is important to mention that fluctuation in multicore fibers are inherently not independent from core to core, while in laser inscribed waveguides the control over the refractive index of individual cores allows independent fluctuations. Our treatment is valid for both cases.

The crux of our approach relies on two fundamental assumptions arising from the small, smooth, and well-behaved variations in the effective propagation constants. First, we model the fiber as a sequence of infinitesimal segments where the supermodes of each segment are provided by first-order perturbation theory on the supermodes of an homogeneous fiber. Second, we treat propagation through the whole fiber as an averaging process for the initial impinging field independently propagating through each of these infinitesimal segments.

In order to calculate the perturbed supermodes, we need to rewrite the coupled mode matrix,

3 $$M(z)={M}_{0}+{M}_{I}(z),$$

in terms of a constant matrix, *M*_0_, and a diagonal perturbation matrix, *M*_1_ = diag(*δβ*_1_(*z*), …, *δβ*_*n*_(*z*), *δβ*_*c*_(*z*)). The constant coupling matrix *M*_0_ has a total of *n* + 1 supermodes^19^. Among these, n − 1 supermodes have zero field component in the central core,

4 $${\hat{S}}_{j}=\frac{1}{\sqrt{n}}\mathop{\sum }\limits_{a=1}^{n}\,{e}^{-i\frac{2\pi }{n}j(a-1)}\,{\hat{e}}_{a}\,for\,j=1,2,\ldots ,n-1,$$

where ${\hat{e}}_{j}$ is the (*n* + 1)-dimensional vector with 1 in its *j*-th component and zero everywhere else. The corresponding *n* − 1 propagation constants are

5 $${\lambda }_{j}=\beta +\{\begin{array}{ll}\,\,\,2\mathop{\sum }\limits_{k=1}^{m-1}\,\,\{{g}_{k}\,\cos \,[\frac{\pi }{m}jk]\}+{g}_{m}\mathrm{\ (}\,-\,{\mathrm{1)}}^{j}, & n=2m,\\ \,\,\,2\mathop{\sum }\limits_{k=1}^{m}\,\,\{{g}_{k}\,\cos \,[\frac{2\pi }{2m+1}jk]\}, & n=2m\,+\,1.\end{array}$$

The two additional supermodes are provided by

6a $${\hat{S}}_{n}=-\,\sin \,\theta \,{\hat{S}}_{0}+\,\cos \,\theta \,{\hat{e}}_{n+1},$$

6b $${\hat{S}}_{n+1}=\,\cos \,\theta \,{\hat{S}}_{0}+\,\sin \,\theta \,{\hat{e}}_{n+1},$$

with propagation constants,

7a $${\lambda }_{n}=({\lambda }_{0}+{\beta }_{c}-\sqrt{{({\lambda }_{0}-{\beta }_{c})}^{2}+4{g}_{c}^{2}n},)/2,$$

7b $${\lambda }_{n+1}=({\lambda }_{0}+{\beta }_{c}+\sqrt{{({\lambda }_{0}-{\beta }_{c})}^{2}+4{g}_{c}^{2}n},)\mathrm{/2.}$$

The difference between these constants yields the Rabi frequency

8 $$\Omega =\sqrt{{({\lambda }_{0}-{\beta }_{c})}^{2}+4n\,{g}_{c}^{2}}\mathrm{.}$$

Additionally, the mixing angle,

9 $$\tan \,\theta =\frac{2\sqrt{n}\,{g}_{c}}{{\lambda }_{0}-{\beta }_{c}+\Omega },$$

parametrizes the whole homogeneous fiber; each different realization of the multicore fiber can be described by its mixing angle. In absence of coupling to the inner core, *g*_*c*_ = 0, these two supermodes are given by the central core mode, ${\hat{S}}_{n}$|_*θ*=0_ = ${\hat{e}}_{n+1}$ with propagation constant *β*_*c*_ and the mode ${\hat{S}}_{n+1}{|}_{\theta =0}={\hat{S}}_{0}$ with propagation constant *λ*_0_. The (*n* + 1)-dimensional vector ${\hat{S}}_{0}$ and the constant *λ*_0_ are defined using Eqs (4) and (5), respectively, with *j* = 0.

### Fluctuation optimization

Now, we include the effects of the small propagation-dependent variations provided by *M*_*I*_(*z*). We use first-order perturbation theory to calculate the unnormalized supermodes for the infinitesimal segment at the propagation distance *z*,

10 $${\overrightarrow{A}}_{j}={\hat{S}}_{j}+\mathop{\sum }\limits_{k\mathrm{=1,\ }k\ne j}^{n+1}\,\frac{{\hat{S}}_{k}^{\dagger }\cdot {M}_{I}\cdot {\hat{S}}_{j}}{{\lambda }_{j}-\lambda k},$$

where *j* = 1, …, *n*, *n* + 1. We aim for isolation between central and external cores after a given propagation distance. For this, we use a target state with equal field amplitude in the external cores and zero in the central core; the uncoupled supermode *Ŝ*_0_ is chosen as initial condition. We look for maximum overlap between our target state and the output after propagation through the fiber. This overlap is quantified by the inverse participation ratio (IPR) between the target mode and the propagation-dependent supermodes,

11 $${\rm{IPR}}[{\hat{S}}_{0},\hat{A}]=\mathop{\sum }\limits_{j=1}^{n+1}\,{|{\hat{S}}_{0}^{\dagger }\cdot {\hat{A}}_{j}|}^{4},$$

where ${\hat{A}}_{j}$ denotes the *j*-th normalized propagation-dependent supermode. A maximum IPR of one is reached when the target state overlaps with a single state of the basis and a minimum of 1/(*n* + 1) when the target state overlaps with all states homogeneously. It is worth noting that the IPR was introduced in the context of localization in disordered quantum system^20,21^. Here, we employ it to quantify the closeness of a preferred mode to an eigenmode of the perturbed system where light localization is feasible. It is cumbersome to calculate the IPR of our target with the propagation-dependent basis as it involves integration over all infinitesimal segments. Instead, we argue that propagation through each infinitesimal segment will induce small changes on the initial field distribution. Thus, instead of calculating the *z*-dependent propagation of an initial field distribution, we calculate the average of the output of that initial distribution through each infinitesimal segment. This allows us to substitute any power of the local variations by the statistical moment of the corresponding power, *δβ*^*m*^ → 〈*δβ*^*m*^〉. For the sake of simplicity, we assume small random variations evenly distributed around zero and keep the leading non-vanishing moment 〈*δβ*^2^〉 to look for ideal crosstalk suppression, IPR[${\hat{S}}_{0}$, $\hat{A}$] = 1. In principle, this leads to a relation between the second moment and the fiber parameters, that we can use to determine the optimal fluctuation amplitude that maximally suppresses crosstalk for a given fiber,

12 $$\begin{array}{rcl}\frac{\langle \delta {\beta }^{2}\rangle }{n\,{\Omega }^{2}} & = & \frac{1-{\cos }^{4}\theta -{\sin }^{4}\theta }{2}\mathrm{\ (6}{\cos }^{4}\theta \,{\sin }^{4}\theta -{\Omega }^{2}{\sin }^{6}\theta \,{{\mathscr{S}}}_{n}\\  &  & -\,{\cos }^{2}\theta {\sin }^{6}\theta -{\Omega }^{2}{\cos }^{6}\theta \,{{\mathscr{S}}}_{n+1}-{\cos }^{6}\theta {\sin }^{2}\theta {)}^{-1},\end{array}$$

where the auxiliary functions are defined by the expression

13 $${{\mathscr{S}}}_{j}=\mathop{\sum }\limits_{k=1}^{n-1}\,\frac{1}{{({\lambda }_{j}-{\lambda }_{k})}^{2}}\,{\rm{for}}\,j=n,n+1.$$

**Figure 2:**
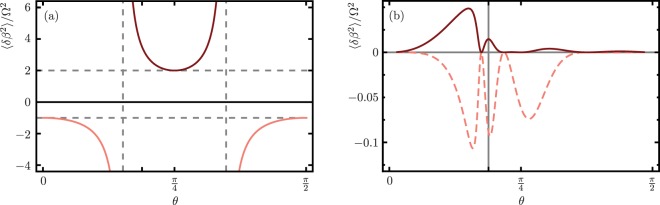
Optimal fluctuation size divided by the Rabi frequency as a function of the mixing angle, *θ*, for a (**a**) two- and (**b**) seven-core system. The vertical solid line marks the mixing core angle for our seven-core system.

**Figure 3:**
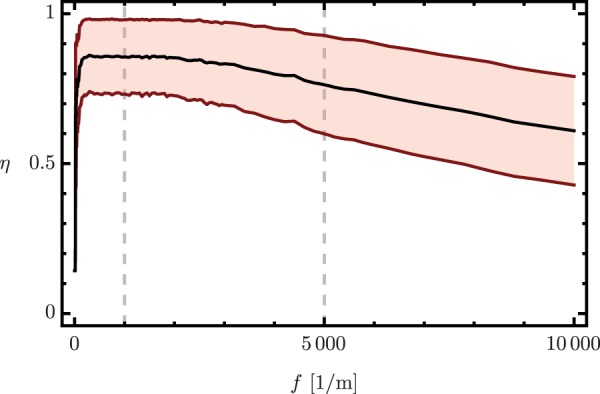
Average and dispersion of irradiance localization in the external cores as a function of the number of random variations per unit length. The vertical gray lines mark the variation frequencies used in our simulations.

For example, random fluctuations with a uniform probability distribution in the range [−*δβ*_max_, *δβ*_max_] have a second moment 〈*δβ*^2^〉 = $\delta {\beta }_{{\rm{\max }}}^{2}$/12 and those with a Gaussian probability distribution have 〈*δβ*^2^〉 = *σ*^2^$\delta {\beta }_{{\rm{\max }}}^{2}$, where *σ* gives the width of the Gaussian distribution and *δβ*_max_ is given by the fluctuation size allowed by the experimental system. Depending on the characteristics of the fiber, sometimes there is a feasible solution for ideal crosstalk suppression; *i.e*., there are positive noise amplitudes producing IPR[${\hat{S}}_{0}$, $\hat{A}$] = 1. Figure 2(a) shows the relation between the scaled second moment and the mixing angle for a two core system, *n* = 1, where viable fluctuation amplitudes for ideal suppression, IPR[${\hat{S}}_{0}$, $\hat{A}$] = 1, are given by the positive branch of the equation. The noise amplitudes that produce ideal crosstalk suppression, IPR = 1, are moderate-noise solutions in the sense that, there, increasing the second momenta of the noise distribution would not lead to more crosstalk suppression. Figure 2(b) shows the result for a seven-core system where ideal suppression is not feasible. We present results for partial suppression, IPR[${\hat{S}}_{0}$, $\hat{A}$] < 1. In this case, our seven-core system, Fig. 3, with mixing angle *θ* = 0.59 rad is shown as a vertical solid line. In the cases where ideal crosstalk suppression is not possible, the inverse participation ratio can still be a useful parameter to find noise parameters that optimize the feasible crosstalk suppression.

Once the maximum feasible crosstalk suppression and corresponding fluctuation amplitude are assessed, we can use coupled mode theory to run a statistical numerical analysis to find the optimal number of variations per length unit that will produce the maximum available crosstalk suppression in a given propagation length, *z*_*c*_. For the sake of simplicity, we focus on crosstalk suppression between the external and inner core. Our figure of merit will be the localization of the field in the external cores given by the ratio between the total irradiance in the external cores with respect to that in all cores,

14 $$\eta (z)=\frac{\mathop{\sum }\limits_{j\mathrm{=1}}^{n}\,|{ {\mathcal E} }_{j}(z{)|}^{2}}{\mathop{\sum }\limits_{j=c\mathrm{,1}}^{n}\,|{ {\mathcal E} }_{j}(z{)|}^{2}}\mathrm{.}$$

Complete localization occurs for *η* = 1, complete transfer to the central core at *η* = 0, and complete delocalization in all cores for *η* = *n*/(*n* + 1). Figure 3 shows our rate of localization in the external cores for a seven-core fiber, *n* = 6, with an uniform random fluctuation distribution with maximum effective propagation constant *δβ*_max_ ≈ 880 rad/m corresponding to a radii variation of 8.5% or a refractive index variation of 3 × 10^−4^. We chose as total propagation distance *z*_*c*_ = 27 × *π*/Ω; an odd integer multiple of the distance providing maximum power transfer to the central core. Figure 3 shows the average (solid black line) and the mean dispersion (light red area) of 5000 independent realizations for up to 10000 variations per meter using coupled mode theory.

### Numerical experiment

**Figure 4:**
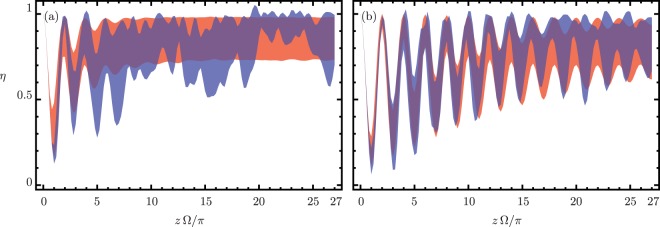
Irradiance localization in the external cores from coupled mode (red region) and finite difference (blue region) propagation for (**a**) optimal and (**b**) sub-optimal variation frequency. The width of colored regions is two standard deviations centered around the average localization.

We use finite difference beam propagation methods to conduct a numerical experiment to try and confirm our predictions. Figure 4 shows the localization parameter versus the propagation distance in terms of the complete delocalization distance, *π*/Ω, for 1000 and 5000 repetitions per meter, in that order, in our seven-core fiber. The coupled mode theory approach and the finite difference methods display slightly different delocalization distances, *π*/Ω = 2323.41 *μ*m and *π*/Ω = 2155.80 *μ*m, respectively. We show the average of 5000 independent exact numerical realizations using coupled mode theory in red. In blue, we show the average of 5 independent realizations propagated using finite difference beam propagation methods. We want to emphasize that the 5000 coupled mode and the 5 finite difference realizations take an average of 3 and 15 hours to compute, in that order. Note that the numerically-predicted optimal number of random variations per unit length, Fig. 4(a), stabilizes in a shorter propagation distance than the sub-optimal value, Fig. 4(b), to similar values of the average irradiance localization.

## Conclusions

We have shown that assuming small, smooth, well-behaved random variations in the core radii of a symmetric multicore fiber allows us to predict, in analytic form, the maximum variation amplitude that will produce crosstalk suppression between its supermodes. We find moderate-noise regions that lead to optimal crosstalk suppression. These are regions where increasing the noise amplitude does not lead to more crosstalk suppression. The analytic maximum variation amplitude, then, helps us numerically define an optimal number of variations per length unit that will produce a stable target suppression at a given propagation length. Our treatment is a simple, low computational resource, design method that is in good agreement with more resource intensive numerical methods and experimental results^5,6^. To the best of our knowledge, this is the first analytic approach to estimate noise parameters in waveguide design.

We find it important to remark that our method is general and valid for any device described by coupled mode theory, independent of the system geometry, nature of the fluctuations or their level of correlation. As long as the supermodes are available, our method provides an analytic optimal fluctuation amplitude for a given level of crosstalk suppression that reduces a stochastic optimization problem in two-parameters into a simple single-parameter exercise.
